# Aerosol Assisted Chemical Vapour Deposition (AACVD) of Zinc dichalcogenoimidodiphosphinate Complexes for the Deposition of Zinc Selenide Thin Films

**DOI:** 10.1002/open.202400295

**Published:** 2024-12-04

**Authors:** Temidayo Oyetunde, Martins O. Omorogie, Paul O'Brien

**Affiliations:** ^1^ Department of Chemical Sciences Faculty of Natural Sciences Redeemer's University P.M.B. 230 Ede 232102 Nigeria; ^2^ School of Chemistry and School of Materials The University of Manchester Oxford Road Manchester M13 9PL UK; ^3^ Water Science and Technology Research Unit African Centre of Excellence for Water and Environmental Research Redeemer's University P.M.B. 230 Ede 232102 Nigeria; ^4^ Chair of Urban Water Systems Engineering, School of Engineering and Design Technical University of Munich Garching 85748 Germany

**Keywords:** AACVD, Cubic zinc selenide, Dichalcogenoimidodiphosphinate, Thin film, Transition metal chalcogenides

## Abstract

Dichalcogenoimidodiphosphinate complexes of zinc [Zn{(EP^i^Pr_2_)_2_N}_2_], [E=Se,Se; S,Se] were synthesized through metathetical reactions from the dichalcogenoimidodiphosphinate ligands [(EE'P^*i*^Pr_2_NH)] (E, E’=Se, Se; S, Se). These complexes were characterized and used as single‐source precursors through Aerosol‐Assisted Chemical Vapour Deposition (AACVD) for the deposition of cubic zinc selenide (ZnSe) films on glass substrates. The deposition temperature occurred at 500 and 525 °C, while the flow rates of the carrier gas was 160 and 240 standard cubic centimetre (sccm). The morphology of the deposited films ranged between dense fibrous network and woven fibres. X‐ray photoelectron spectroscopy (XPS) confirmed the presence of the constituent elements in zinc selenide.

## Graphical abstract

Zinc complexes have been prepared from dichalcogenoimidodiphosphinate ligands. Aerosol‐assisted chemical vapour deposition (AACVD) of these single‐source precursors deposit thin films of cubic zinc selenide at the different deposition temperatures and flow rates of the carrier gas. Hetero‐dichalcogeno complex of zinc exclusively deposit zinc selenide

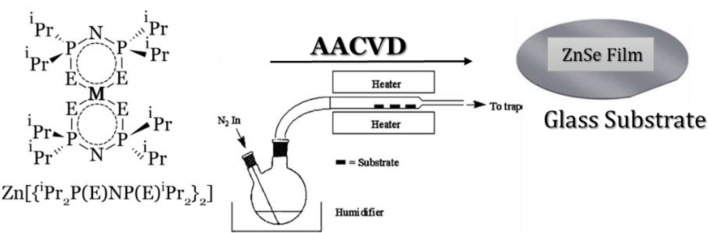

## 1 Introduction

Transition metal chalcogenides (TMCs) represent a class of materials that possess interesting properties and diverse applications in different areas, which has stimulated increasing intense research in recent years. Some of their applications include supercapacitors, water splitting photocatalysis, fuel cells, solar cells, sensors, and photovoltaics.[^1^, ^2^, ^3^, ^4^, ^5^, ^6^] From the II–VI family of semiconductors, zinc chalcogenides are wide band‐gap semiconductors which have been studied due to certain characteristic features.[^6^, ^7^, ^8^] These include: thermoelectric,[^9^, ^10^] photo‐catalysis,^[11]^ opto‐electronic,^[10]^ electronic^[12]^ and thermal properties.^[13]^ As a member of the II–VI family, zinc selenide (ZnSe) is an *n‐*type semiconductor with a direct band‐gap of 2.7 eV[^14^, ^15^, ^16^] with optical properties.^[17]^ Hence, ZnSe has wide applications in devices such as lasers, optical storage, colour displays, light emitting diodes (LEDs), transistors and biomedicine.[^14^, ^17^, ^18^] Using physical and chemical methodologies, ZnSe can be synthesised both in the form of thin films and powder.^[17]^ Some of the reported deposition techniques for the thin films of ZnSe include: electrodeposition, chemical vapour deposition, thermal and vacuum evaporation, magnetron sputtering and molecular beam epitaxy.^[19]^ From electrodeposition, zinc sulphate and selenium dioxide were the starting materials for the deposition of ZnSe thin films on fluorine‐doped tin oxide coated glass substrates.^[19]^ From hydrothermal synthesis, ZnSe was prepared from zinc chloride and sodium selenite in deionized water, followed by oven drying at 140 °C^20^. By using chemical bath deposition involving zinc acetate dihydrate and sodium selenosulfate at a constant p^H^, thin films of ZnSe were deposited on glass substrates.^[14]^ On fluorine‐doped tin oxide substrates, ZnSe thin films were deposited from zinc tetraoxosulphate(VI) heptahydrate and selenium(IV) oxide using electrochemical deposition technique.^[21]^ Using a mixture of zinc tetraoxosulphate(VI) heptahydrate and selenium powder having cobalt as dopant, the photovoltaic properties of the deposited ZnSe thin films on fluorine‐doped tin oxide substrate were investigated using electrochemical deposition technique.^[22]^ From ZnSe powder, ZnSe thin films were deposited on glass substrates by electron beam evaporation.^[16]^

Among the deposition techniques, aerosol‐assisted chemical vapour deposition (AACVD) is a class of CVD which is cheap with simple installation, and can be operated at reduced pressure to deposit high purity and reproducible thin films.^[23]^ AACVD requires the solution of the precursor as its feed is transported through an aerosol generated by a humidifier. Hence, the solubility of the precursor in a solvent is a fundamental requirement in AACVD. This technique affords control over stoichiometry together with circumventing impurities and unwanted particles in the films to be deposited, thereby producing tailor‐made thin films.[^24^, ^25^]

Single‐source precursors are inorganic complexes containing both the metal and chalcogen in a single molecule used for the deposition process. Single‐source precursor approach affords reduced temperature and environmentally‐friendly syntheses which are economically cheap with minimal environmental hazards. Through ligand design, the precursor can be tailor‐made with desired targeted properties *e. g*. purity, atom‐efficiency, volatility, *etc*. Single‐source precursor route provides a steady precursor feedstock maintenance, stoichiometric control and also circumvents the formation of pre‐reactions. Both the decomposition mechanism and temperature can be monitored through the precursor design which provides an elimination pathway. Some of the recently reported single‐source precursors for metal selenides include complexes such as: diselenoimidophosphinate, diselenophosphinate, diselenophosphate, diselenocarbamate, selenourea, selenoether, selenolate and selenocarboxylate.[^26^, ^27^, ^28^]

As bidentate ligands, the dichalcogenoimidodiphosphinate ligands [(EE'P_2_R_4_NH)] (E, E’=S, S; Se, Se; S, Se; Te, Te; R=^*i*^Pr, Ph) can coordinate with transition metals to form various inorganic complexes having ring structures.[^29^, ^30^, ^31^, ^32^, ^33^, ^34^] Besides the P−N−P structure, the ligand also has two organic moieties and two phosphorus atoms (connected to the chalcogenide atoms). This entity coordinates with the metal atom to produce ring‐like inorganic coordination compounds.

Using low pressure CVD (LPCVD), zinc selenide thin films were deposited from the precursor [Zn{(SePR_2_)_2_N}_2_] (R=Ph, ^i^Pr).[^35^, ^36^] From zinc *bis* [methyl(n‐hexyl)‐diselenocarbamato] complex as the precursor, thin films of cubic ZnSe were deposited on glass substrates between 30 and 90 minutes using low pressure metal organic chemical vapour deposition (LP‐MOCVD).^[37]^ Using MOCVD on different substrates, both the cubic and hexagonal phase of ZnSe thin films were observed (based on the deposition temperature) from asymmetric diselenocarbamate complexes of zinc: Zn(N‐ethylbutyldiselenocarbamate)_2_ and Zn(2‐ethylpiperidinediselenocarbamate)_2_.^[38]^ When zinc *bis* (diethyldiselenocarbamato) complex was employed as the precursor, hexagonal thin films of ZnSe were deposited on glass substrates by AACVD.^[39]^

To the best of our knowledge, there is/are no report(s) yet on the use of zinc dichalcogenoimidodiphosphinate complexes as single‐source precursors to deposit thin films of ZnSe on substrates using AACVD.

In this paper, we present the deposition of thin films of cubic zinc selenide by aerosol‐assisted CVD (AACVD) on glass substrates from both the diseleno‐ and thioseleno‐imidodiphosphinate complexes of zinc, [Zn{(EP^i^Pr_2_)_2_N}_2_] (E=Se, Se; E=S, Se). Deposition temperatures were at 500 and 525 °C, while the flow rates of argon were 160 and 240 standard cubic centimetre (sccm).The deposited films were characterized by X‐ray diffraction (XRD), scanning electron microscopy (SEM), energy dispersive analysis of X‐ray (EDAX) and X‐ray photoelectron spectroscopy (XPS).

## Experimental Section

### Materials

Under a dry inert atmosphere of nitrogen, all experiments were performed using a standard double manifold Schlenk line connected to a vacuum pump. Chemicals and reagents were obtained from Sigma‐Aldrich and used without any further processing. Through distillation or molecular sieves storage, all solvents were obtained dried and used throughout the experiments.

### Synthesis of Ligands^[32]^

#### Synthesis of [^i^Pr_2_P(S)NHP(Se)^i^Pr_2_]^[32]^

The ligand was synthesised as described in the literature.

#### Synthesis of [^i^Pr_2_P(Se)NHP(Se)^i^Pr_2_]^[32]^

The ligand was synthesised as described in the literature.

### Synthesis of Complexes

#### Synthesis of Zn[(SP^i^Pr_2_)(SeP^i^Pr_2_)N]_2_(1)

Thioselenoimidodiphosphinate ligand (5.9 g, 16 mmol) was dissolved in dry methanol, stirred for 30 min and sodium methoxide NaOMe (0.86 g, 16 mmol) added. After 20 min, zinc chloride ZnCl_2_ (1.11 g, 8 mmol) was added and gave a cloudy white suspension. The reaction mixture continued for 4 h which gave a cloudy white suspension. This was filtered and followed by solvent removal under vacuum, which gave a white powder. Yield: 2.4 g (62 %). Microanalysis calculated for C_24_H_56_N_2_P_4_S_2_Se_2_Zn: C, 36.75; H, 7.20; N, 3.57; P, 15.81; S, 8.16; Zn, 8.35. Found: C, 36.68; H, 7.26; N, 3.50; P, 15.98; S, 7.88; Zn, 8.34. ^1^H NMR, (δ, CDCl_3_, 400 MHz): δ=1.19 (m, 24H, 8×CH(CH_3_)_2_); 1.54 (m, 24H, 8×CH(CH_3_)_2_); ^13^C NMR: δ=16.76, 32.27, 77.08 ppm; ^31^P {^1^H} NMR: 57.07, 65.55 ppm.

#### Synthesis of Zn[(SeP^i^Pr_2_)_2_N]_2_(2)

Diselenoimidodiphosphinate ligand (4.75 g, 11.67 mmol) was dissolved in dry MeOH, stirred for 30 min and NaOMe (0.63 g, 11.67 mmol) was added. An orange/yellow colouration was observed followed by gentle heating which gave a clear transparent dark‐brown solution. ZnCl_2_ (0.80 g, 5.83 mmol) was added and resulted in a cloudy white suspension immediately. The reaction was continued for 3 h and a cloudy white suspension was obtained, filtered and dissolved in hot dichloromethane. The resulting solution was filtered to remove excess reactant followed by solvent removal under vacuum. A white powder was obtained. Yield: 3.0 g (59 %). Elemental analysis calculated for C_24_H_56_N_2_P_4_Se_4_Zn: C, 32.82; H, 6.43; N, 3.19; P, 14.12; Zn, 7.45 %. Found: C, 32.72; H, 6.46; N, 3.10; P, 13.74; Zn, 7.14 %. ^1^H NMR, (δ, CDCl_3_, 400 MHz): δ=1.15 (m, 24H, 8×CH(CH_3_)_2_); 1.56 (m, 24H, 8×CH(CH_3_)_2_); ^13^C NMR: δ=17.10, 32.48 and 76.70 ppm; ^31^P {^1^H} NMR: 57.07, 65.55 ppm.

### Characterization of the Complexes

Elemental analyses of the complexes were done at the Microanalytical unit in the School of Chemistry, University of Manchester, United Kingdom. Under nitrogen and a heating rate of 10 °C min^−1^, thermogravimetric analyses (TGA) were performed using Seiko SSC5200/S220TG/DTA model instrument. Using either CDCl_3_ or d_8_‐toluene as solvent, NMR spectra were recorded on a Bruker Avance (III) 400 MHz FT‐NMR spectrometer. ^1^H NMR spectra were reported with reference to tetramethylsilane, Me_4_Si. ^31^P NMR spectra were reported relative to H_3_PO_4_.

### Aerosol Assisted Chemical Vapour Deposition (AACVD)

In a two‐necked 100 mL round‐bottom flask, 0.2 g of the precursor was dissolved in 20 mL of toluene for each deposition experiment. Through a gas inlet connected to the flask, the carrier gas (argon) entered the solution for the rapid transport of the aerosol. In a Carbolite furnace, the flask was connected to the reactor tube through reinforced tubing. Being controlled by a Platon flow gauge, the carrier gas flowed at the rate of either 160 or 240 sccm during each deposition experiment. Glass substrates (*ca*. 1×3 cm) were placed inside the reactor tube, located in a Carbolite furnace. The solution of the precursor in the flask was kept in a water bath above the piezoelectric modulator of a PIFCO ultrasonic humidifier (Model No. 1077). From the precursor, the generated aerosol droplets were transported into the hot wall deposition zone of the reactor by the carrier gas. Evaporation of both the solvent and the precursor occurred, while the precursor vapour reached the heated substrate surface. This was where thermally induced reactions and film deposition occurred. Each deposition experiment was done between 90 min and 120 min.

### Thin Films Characterization

A monochromated Cu−Kα radiation on a Bruker AXS D8 diffractometer was used for the X‐ray diffraction (XRD) analyses. In a step size of 0.05 together with a count rate of 9 s, samples were scanned from 10 to 85° after being mounted flat. The obtained diffractogram patterns were then compared to the documented patterns in the ICDD index. Before scanning electron microscopy (SEM) was done, samples were carbon‐coated to remove charging of the sample by the electron beam, using Edward's coating system E306 A. SEM was done on a Philips XL30 FEG SEM. Energy dispersive analyses of X‐rays (EDAX) were performed on a DX4 instrument. A Kratos Axis Ultra spectrometer fitted with a monochromated Al K_α_ X‐ray source and analyser pass energy of either wide or narrow scans (80 eV or 20 eV) was used for the X‐ray photoelectron spectroscopy (XPS) measurements. Exposure of the photoemitting surface to low energy electrons in a magnetic immersion lens system (Kratos Ltd.) provided a homogeneous charge neutralisation of the surface. The base pressure of the system was 5×10^−10^ mBar. After subtracting a Shirley background, spectra were analysed to obtain precise peak positions by fitting the peaks using a mixed Gaussian/Lorenzian (30/70) line shape. While fitting the peaks, confinement of the spin orbit split components produced similar line width, elemental spin orbit energy separations and theoretical spin orbital area ratios. Theoretical Scofield elemental sensitivities and recorded spectrometer transmission functions produced the quantitative analyses. At 285 eV binding energy (BE), C1s adventitious contamination peaks were the references for all the photoelectron binding energies. The analyser was calibrated using the following elemental references: Au 4f_7/2_ (83.98 eV BE), Ag 3d_5/2_ (368.26 eV BE) and Cu 2p_3/2_ (932.67 eV BE).

## 2 Results and Discussion

**Scheme 1:**
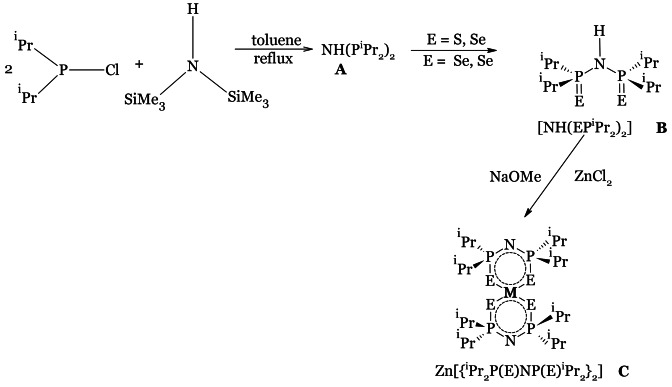
Reaction scheme for the synthesis of dichalcogenoimidodiphosphinate ligands and zinc complexes.

The synthesis of the ligands^[32]^ and complexes^[32]^ occurred through a multi‐step procedure in one‐pot according to the equation of reaction in scheme 1 below. Chlorodiisopropylphosphine and hexamethyldisilazane were refluxed to produce an intermediate [NH(P^*i*^Pr_2_)_2_] (**A**), followed by the addition of selenium in stoichiometric amount or an equimolar amount of sulphur and selenium to produce the ligand [NH(EP^*i*^Pr_2_)_2_] (**B**). Using a base, the acidic proton in the ligand was removed followed by the complexation reaction with ZnCl_2_ to produce the complexes (**C**).

From the IR spectra analysis, some of the selected absorption bands from complex (**1**)(Figure S1) include: ν(P−Se) 428 (s); ν(P−S) 475 (s); ν(Zn−S) 629, 679 (s); ν(P−N−P) 1227, 768 (s); ν(Zn−Se) 887 (s); ν(C−H) methyl 1361, 1384 (s); ν(C−H) CH_2_ 1458 (m); ν(C−H) stretch 3000–2840 (w). From complex (**2**) (Figure S2), some of the selected absorption bands include: ν(P−Se) 428 (s); ν(P−N−P) 1226, 760 (s); ν(Zn−Se) 887 (s); ν(C−H) methyl 1361, 1384 (s); ν(C−H) CH_2_ 1458 (m); ν(C−H) stretch 3000–2840 (w). For complex (**1**), the ^1^H NMR spectra (Figure S3) indicated that the *methyl protons* appeared as multiplets at 1.19 ppm, while the *methylene protons* as multiplets were also observed at 1.54 ppm. The ^13^C NMR spectra (Figure S4) for complex (**1**) indicated that the *methyl carbons* occurred at 16.76 ppm, while the *methylene carbons* were seen at 32.27 ppm. The ^31^P {^1^H} NMR spectra (Figure S5) for complex (**1**) two phosphorus peaks at 57.07 and 65.55 ppm respectively. From the ^1^H NMR spectra (Figure S6) for complex (**2**), the multiplets *methyl protons* were observed at 1.15 ppm, while the *methylene protons* appeared as multiplets at 1.56 ppm. The ^13^C NMR spectra (Figure S7) for complex (**2**) revealed the appearance of *methyl carbons* at 17.10 ppm, while the *methylene carbons* were observed at 32.48 ppm. The ^31^P {^1^H} NMR spectra (Figure S8) for complex (**2**) showed that two phosphorus peaks occurred at 57.07 and 65.55 ppm respectively.

### 2.1 AACVD of [Zn{^i^Pr_2_P(S)NP(Se)^i^Pr_2_}_2_] (Zinc Thioselenoimidodiphosphinate)(1)

**Figure 1:**
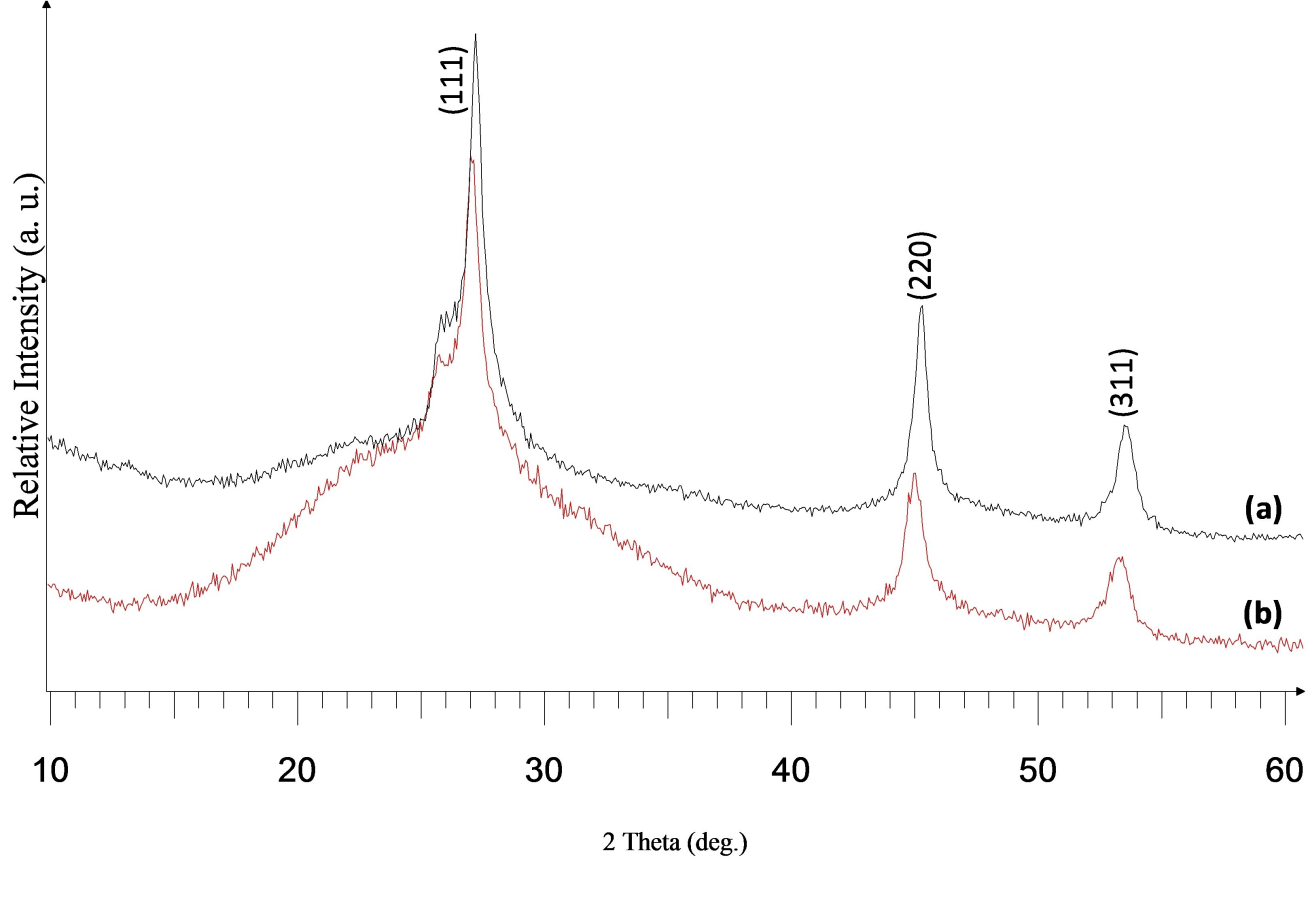
XRD of cubic ZnSe deposited from (**1**) at (a) 525 °C and (b) 500 °C, both at 240 sccm.

**Figure 2:**
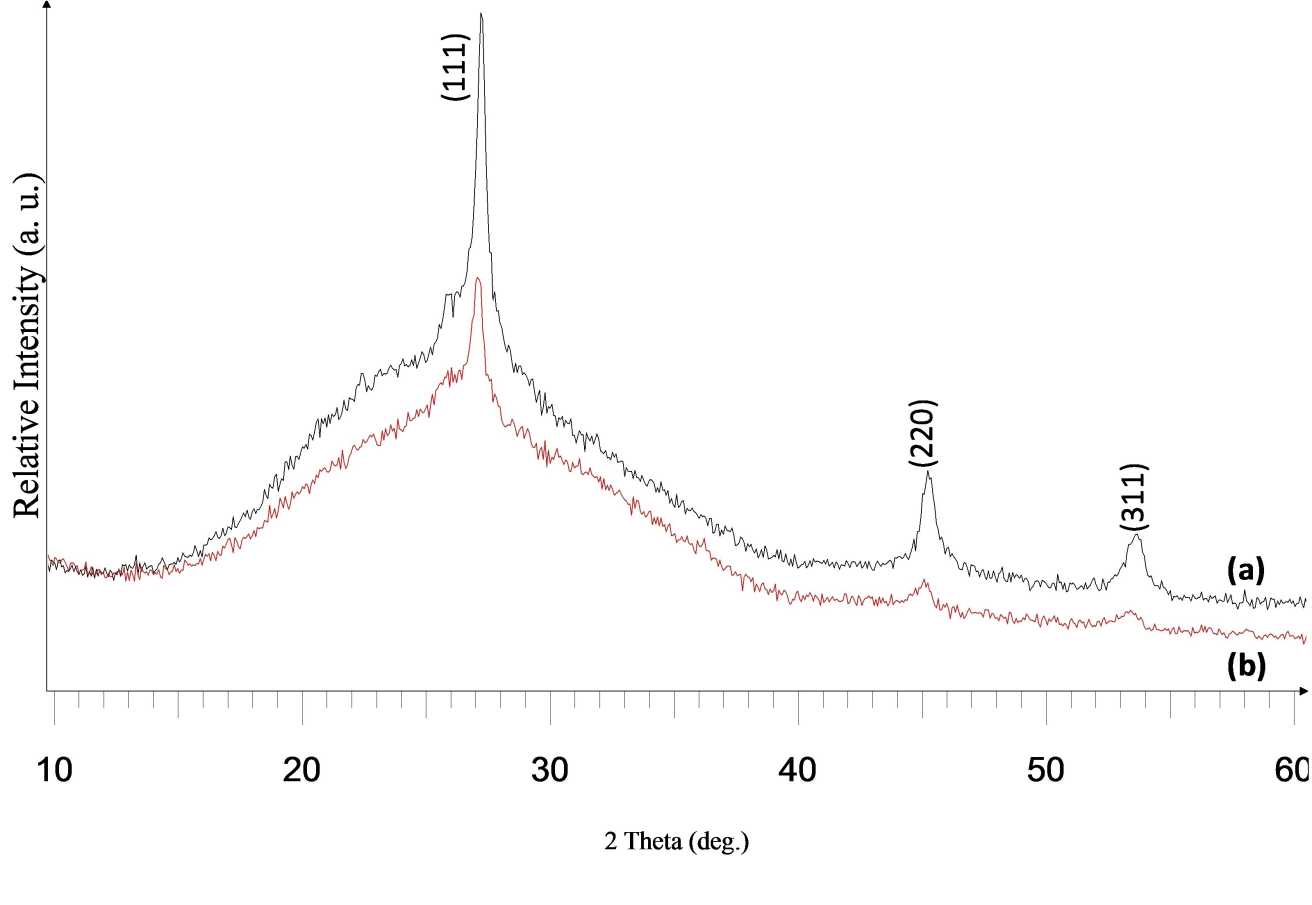
XRD of cubic ZnSe deposited from (**1**) at (a) 525 °C and (b) 500 °C, both at 160 sccm.

Thermogravimetric analysis (TGA) of this precursor clearly revealed its suitability for deposition with a single step decomposition between 267 °C and 387 °C at 99 % (Figure S9, (ESI)). Deposition was carried out at substrate temperatures of 500 °C and 525 °C, with argon flow rates of 160 and 240 sccm onto glass substrates. The optimum temperature appeared to be within this range as there was no deposition below 500 °C. The deposited films were yellow at the flow rate of 240 sccm, while brown films were seen for the films at 160 sccm. All the films were well adherent to the glass surface. X‐ray diffraction (XRD) pattern of the as‐deposited film indicated that the cubic zinc selenide (ICDD 00–037‐1463) were deposited at both 500 °C and 525 °C (Figures 1 and 2). The crystallinity of the as‐deposited films was improved at 525 °C, as revealed by the intensity of the XRPD pattern. At the higher deposition temperature of 525 °C, there was a corresponding increase observed in the intensity of the peaks. This observation is in agreement with a previous report.^[38]^

Previously, there was a report on the growth of hexagonal ZnSe from [Zn{(SePPh_2_)_2_N_2_}]^[35]^ and [Zn{(SeP^i^Pr_2_)_2_N_2_}]^[36]^ by LP‐MOCVD. However, cubic ZnSe was deposited from [Zn(Se_2_CNMe^*n*^Hex)_2_]^[40]^ and [Zn(t‐Bu_2_PSeNR)_2_] (R=^i^Pr, c‐C_6_H_11_)^[15]^ both having the preferred (111) orientation.

Previous deposition studies on [Ag(^i^Pr_2_P(S) NP(Se)^i^Pr_2_)]_3_ precursor using AACVD and low‐pressure CVD (LPCVD) produced Ag_2_Se thin films,^[41]^ while AACVD investigations on Ni[{(SeP^*i*^Pr_2_)(TeP^*i*^Pr_2_)N}_2_] resulted in hexagonal nickel telluride.^[42]^ Deposition experiments on Ni[{(SP^*i*^Pr_2_)(SeP^*i*^Pr_2_)N}_2_] complex as a precursor deposited either nickel phosphide or nickel selenide based on the deposition parameters^42^. The enlargement of the PNP bond angle due to the flexible structure of the isopropyl groups_[42]_ might have favoured the formation of the higher chalcogenide as the final product as observed in the AACVD studies of complex (**1**).

**Figure 3:**
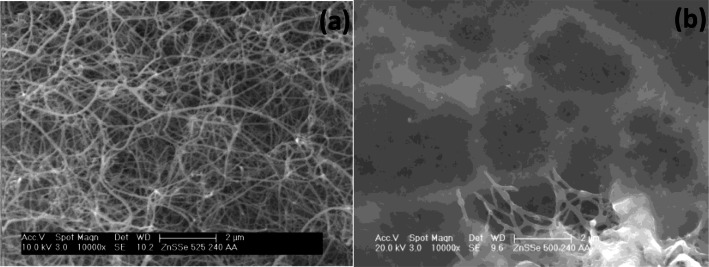
SEM images of cubic zinc selenide deposited from (**1**) at (a) 525 °C and (b) 500 °C at 240 sccm.

**Figure 4:**
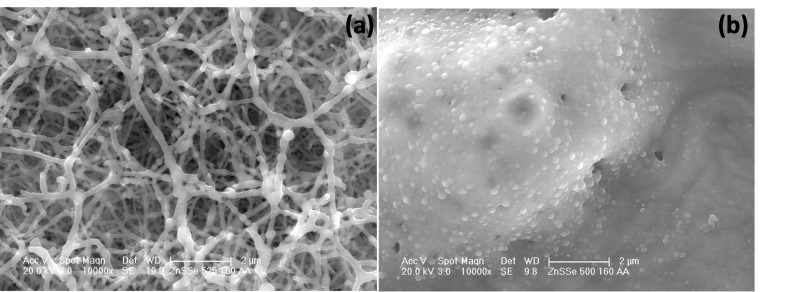
SEM images of cubic zinc selenide deposited from (**1**) at (a) 525 °C and (b) 500 °C at 160 sccm.

Scanning electron microscopy (SEM) studies of the as‐deposited films at 240 sccm revealed an interwoven fibrous network at 525 °C (Figure 3a), while a less pronounced network of fibres was observed for the film at 500 °C (Figure 3b). At 160 sccm, the deposited film at 525 °C had morphology of woven fibres, though seemed to be slightly disordered when compared with the film at 240 sccm (Figure 4a). A tiny globular morphology was observed for the deposited film at 500 °C 160 sccm (Figure 4b).

Energy dispersive X‐Ray analysis (EDAX) of the cubic ZnSe films indicated that the films were rich in Zinc, though with phosphorus contamination. A near stoichiometric ratio of 1 : 1 for Zn and Se was observed for all the films.

### 2.2 AACVD of [Zn{(SeP^i^Pr_2_)_2_N}_2_] (Zinc Diselenoimidodiphosphinate)(2)

**Figure 5:**
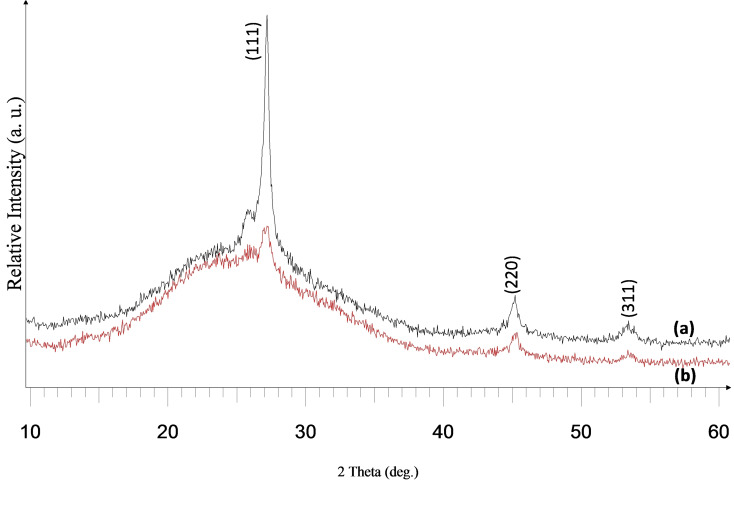
XRD pattern of cubic zinc selenide deposited from (**2**) at (a) 525 °C and (b) 500 °C at 240 sccm.

**Figure 6:**
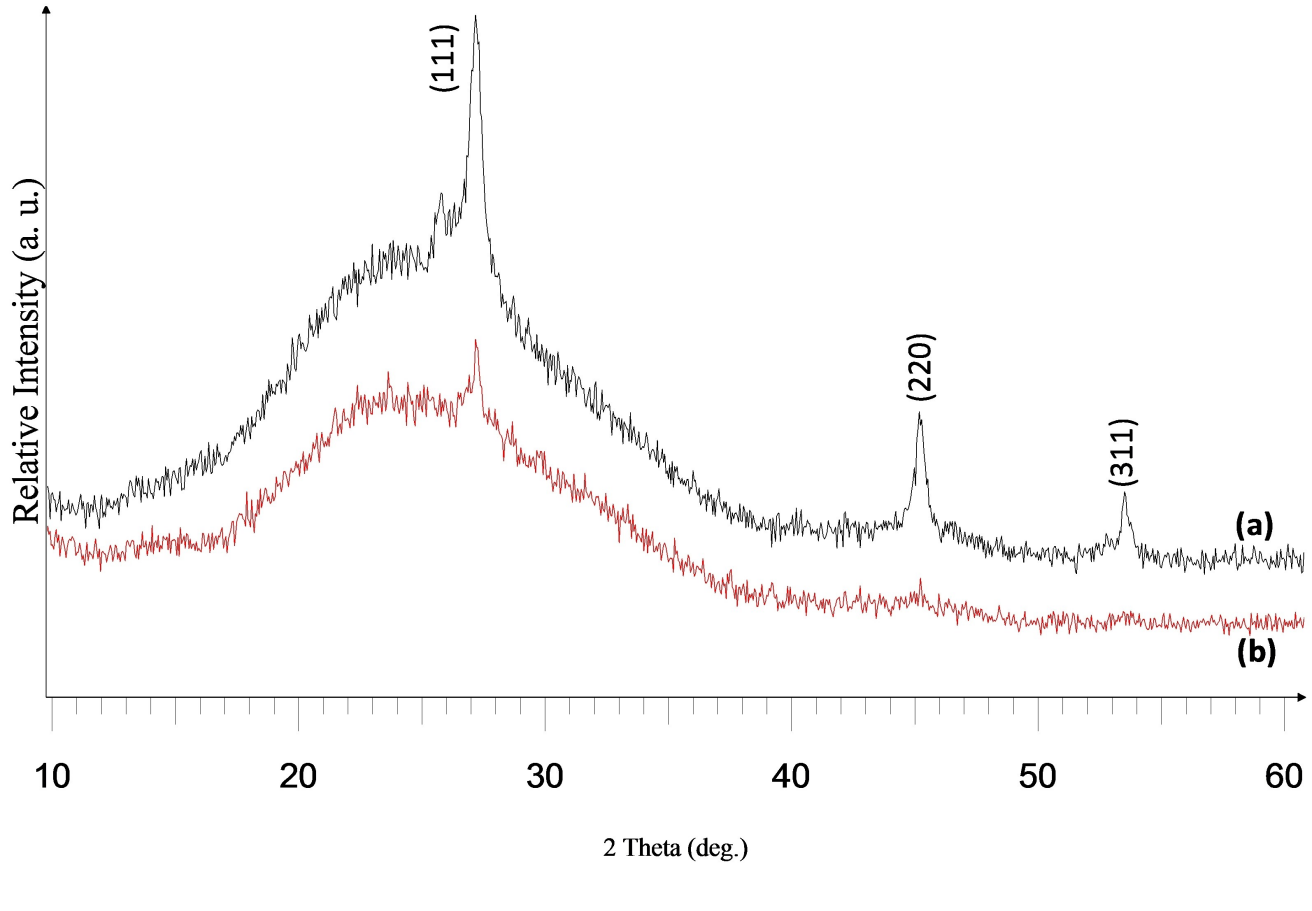
XRD pattern of cubic zinc selenide deposited from (**2**) at (a) 525 °C and (b) 500 °C at 160 sccm.

This precursor was quite suitable for deposition studies as revealed by the thermogravimetric analysis (TGA) with a single step decomposition between 277 and 395 °C at 99 % (Figure S10, ESI). Deposition was done at substrate temperatures of 500 and 525 °C at argon flow rates of 160 and 240 sccm onto glass substrates. All the deposited films were yellow, transparent and well adherent to the glass surface at both the deposition temperatures and flow rates. The X‐Ray diffraction (XRD) pattern of the as‐deposited films showed the exclusive deposition of cubic ZnSe (ICDD 00–037‐1463) (Figure 5 and 6)

**Figure 7:**
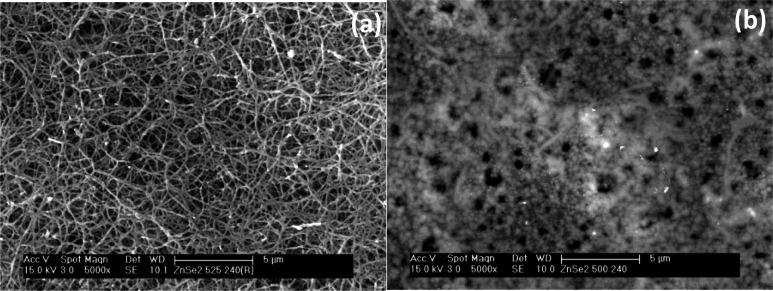
SEM images of cubic zinc selenide deposited from (**2**) at (a) 525 °C and (b) 500 °C at 240 sccm.

Also, the deposited films had improved crystallinity as shown by the increased intensity of the XRD pattern at 525 °C. Scanning electron microscopy (SEM) analysis of the films at 240 sccm revealed a dense fibrous network structure at 525 °C, while porous particles containing thin fibres were seen at 500 °C (Figure 7a and 7b).

**Figure 8:**
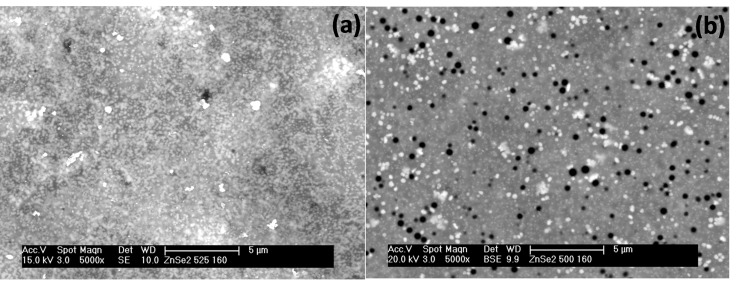
SEM images of cubic zinc selenide deposited from (**2**) at (a) 525 °C and (b) 500 °C at 160 sccm.

However, at the lower flow rate (160 sccm), a marked change in the morphology of the films was vividly seen. The as‐deposited film at 525 °C consisted of random particles, while porous particles were the morphology of the film at 500 °C (Figure 8a and 8b).

Energy Dispersive Analysis of X‐rays (EDAX) indicated the presence of zinc and selenium for all the films alongside with phosphorus. The deposited films at 525 °C (240 and 160 sccm) contained higher amounts of zinc and selenium when compared with that obtained at 500 °C.

### 2.3 X‐Ray Photoelectron Spectroscopy (XPS) Studies

**Figure 9:**
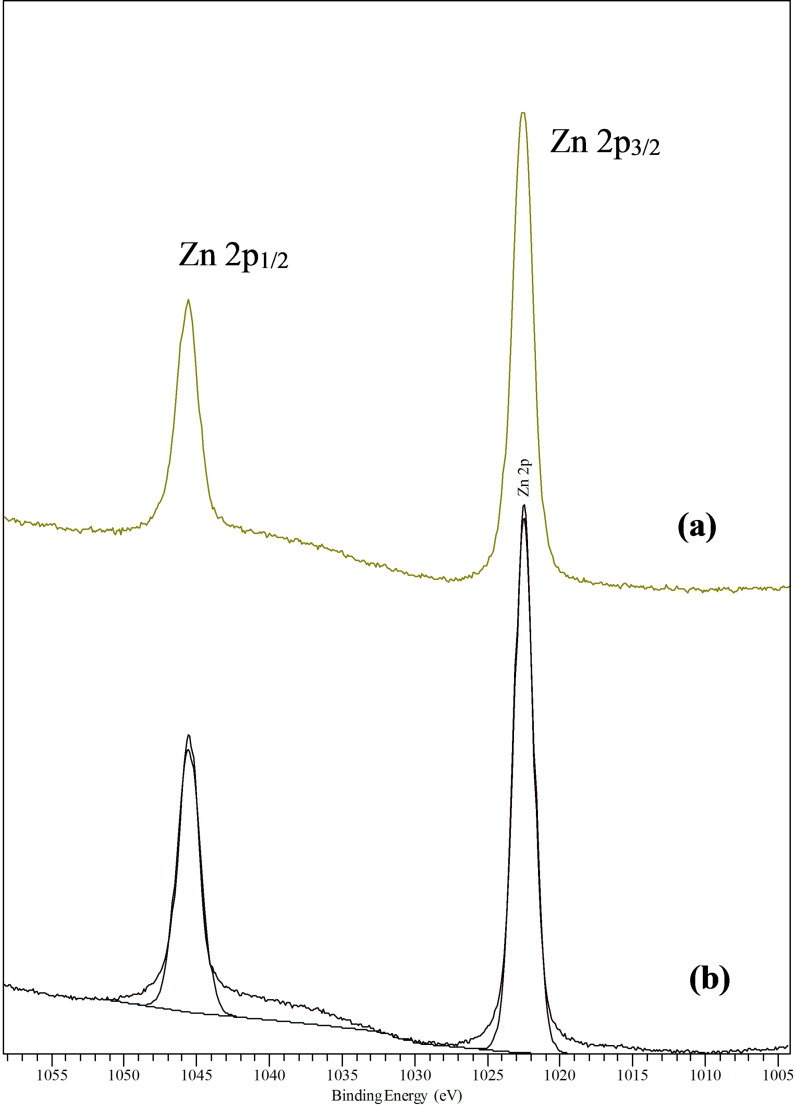
XPS of Zn 2p peaks of zinc selenide deposited from (a) [Zn{^i^Pr_2_P(Se)NP(S)^i^Pr_2_}_2_] (**1**) and (b) [Zn{^i^Pr_2_P(Se)NP(Se)^i^Pr_2_}_2_] (**2**) precursor.

The surface atomic coverage and chemical shifts in the deposited films were determined using XPS. For the films grown from the two precursors at both deposition temperatures and flow rates, the Zn 2p spectra consisted of symmetrical narrow peaks corresponding to 2p_3/2_ and 2p_1/2_spin split orbitals. The XPS spectra showed a Zn 2p_3/2_ peak at 1022.5 eV (Figure 9a and 9b). This corresponded to either ZnO or ZnSe. The presence of oxide was inevitable because of handling of the sample in an open atmosphere. Also, water vapour and other gases in the atmosphere might be the source of oxygen contamination.

**Figure 10:**
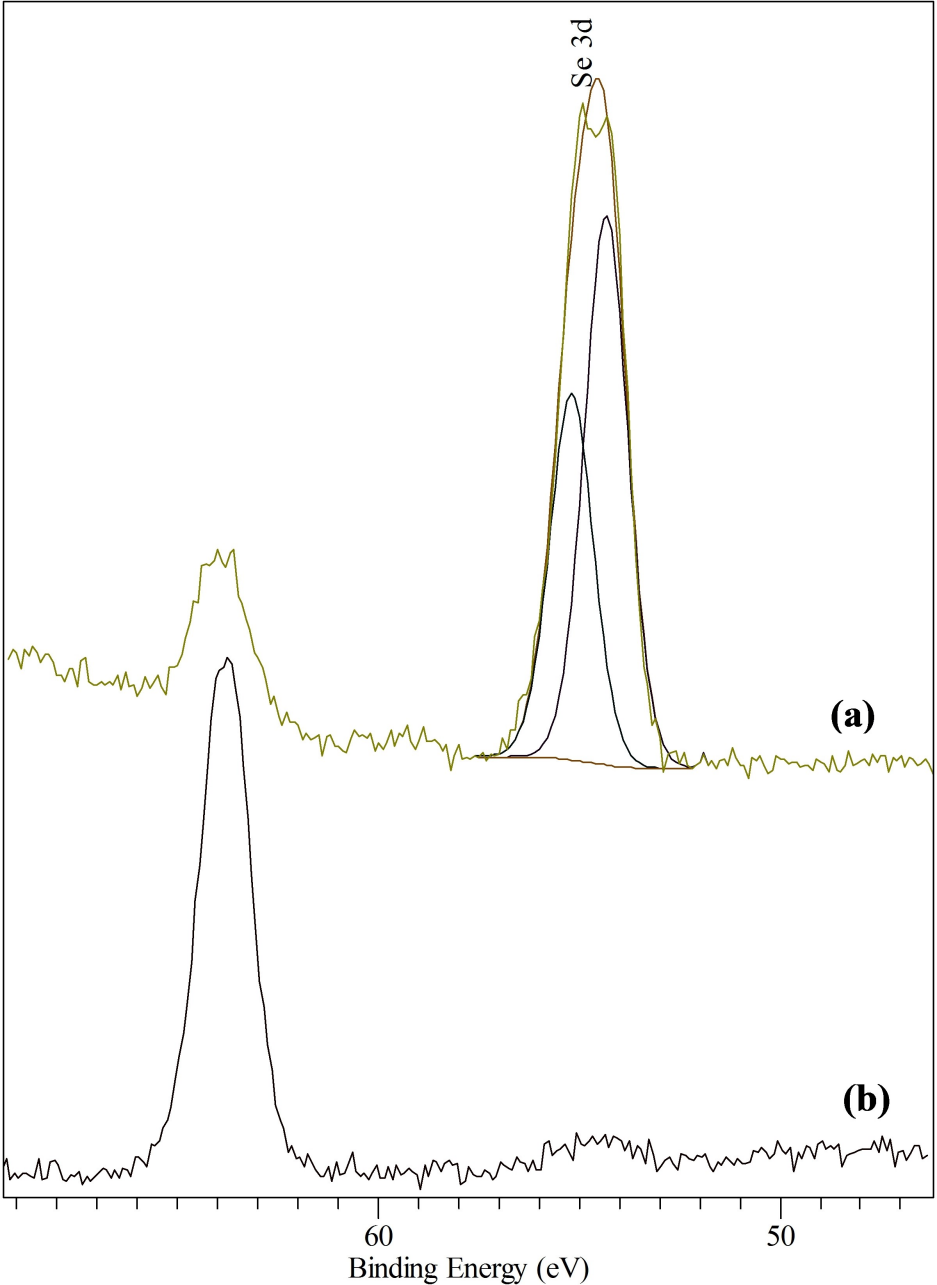
XPS of Se 3d peaks of Zinc selenide deposited from (a) [Zn{^i^Pr_2_P(Se)NP(S)^i^Pr_2_}_2_] (**1**) precursor and (b) [Zn{^i^Pr_2_P(Se)NP(Se)^i^Pr_2_}_2_] (**2**) precursor. Se peak was not seen for (**2**).

The obtained values for the binding energies of Zn 2p_3/2_ and Se 3d seemed to be in agreement with previously reported values of 1021.6 eV and 54.2 eV^[33]^ and 1020.6 eV and 53.3 eV[^12^, ^44^] for bulk crystalline zinc selenide. The Se 3d peak revealed a doublet having a dominant component at 54.1 eV and a smaller one centered at about 55.1 eV respectively (Figure 10). However, no Se peak was seen for complex (**2**) (Figure 10b). The reason (s) for this observation remained unclear.

The Se 3d spectra exhibited a doublet indicative of ZnSe and free Se at 54.1 eV and 55.1 eV respectively. This occurs when atoms of an element are present in different oxidation states, thereby resulting in core‐level peaks having different binding energies.^[44]^ Consequently, increase in the oxidation state of an atom results in corresponding increase in the binding energy. Hence, the Se 3d peaks at 54.1 eV and 55.1 eV could be assigned to be zinc selenide and free selenium respectively. Oxidised Se was not seen to be present because a higher BE Se 3d peak should be seen in this case. The absence of Se 3d peak for the ZnSe_2_ precursor indicated that no Se was present in the top 60 Å on this surface (the maximum escape depth for photoelectrons at XPS in this range). This does not rule out the presence of selenide bulk species.

## 3 Conclusions

From the single‐source precursors of zinc, [Zn{(EP^i^Pr_2_)_2_N}_2_], [E=Se,Se; S,Se] thin films of cubic zinc selenide were deposited on glass substrates through AACVD. Thermogravimetric analysis (TGA) of the complexes revealed a single‐step decomposition between 267 and 395 °C. An increase in the decomposition temperature similarly increased the morphology of the deposited films. Although X‐ray photoelectron spectroscopy (XPS) indicated the electronic states of the constituent elements in the deposited films, no selenium peak was observed for the zinc diseleno precursor. Future work on this will involve the investigation of different synthetic routes for the deposition of zinc thioseleno as a nanomaterial for possible applications.

## Conflict of Interests

The authors declare no conflict of interest.

## Supporting information

As a service to our authors and readers, this journal provides supporting information supplied by the authors. Such materials are peer reviewed and may be re‐organized for online delivery, but are not copy‐edited or typeset. Technical support issues arising from supporting information (other than missing files) should be addressed to the authors.

Supporting Information

## Data Availability

The data that support the findings of this study are available from the corresponding author upon reasonable request.
