# Supplementary material for: Aerosol Assisted Chemical Vapour Deposition (AACVD) of Zinc dichalcogenoimidodiphosphinate Complexes for the Deposition of Zinc Selenide Thin Films
Source: ChemistryOpen. 2024 Dec 4;14(6):e202400295. doi: 10.1002/open.202400295 (PMC12138053; doi:10.1002/open.202400295)
Supplement: Supplementary file 1 — Supporting Information [file OPEN-14-e202400295-s001.pdf]

ChemistryOpen

Supporting Information

Aerosol Assisted Chemical Vapour Deposition (AACVD) of Zinc dichalcogenoimidodiphosphinate Complexes for the Deposition of Zinc Selenide Thin Films

Temidayo Oyetunde,* Martins O. Omorogie,* and Paul O'Brien

Electronic Supplementary Information

Aerosol Assisted Chemical Vapour Deposition (AACVD) of Zinc dichalcogenoimidodiphosphinate Complexes for the Deposition of Zinc Selenide Thin Films

Temidayo Oyetunde,[a,b*] Martins O. Omorogie,[a,c*] Paul O'Brien[b,†]

[a]Department of Chemical Sciences, Faculty of Natural Sciences, Redeemer's University, P.M.B. 230, Ede, 232102, Nigeria.

[b]School of Chemistry and School of Materials, The University of Manchester, Oxford Road, Manchester, M13 9PL, UK.

[c]Water Science and Technology Research Unit, African Centre of Excellence for Water and Environmental Research, Redeemer's University, P.M.B. 230, Ede, 232102, Nigeria.

[d]Chair of Urban Water Systems Engineering, School of Engineering and Design, Technical University of Munich, Garching 85748, Germany.

Corresponding Authors: oyetundetemi@run.edu. ng; omorogiem@run.edu.ng; mo.omorogie@tum.de; dromorogiemoon@gmail.com

† *This paper is in special honour of late Professor Paul O'Brien, who passed away during the preparation of this manuscript.*

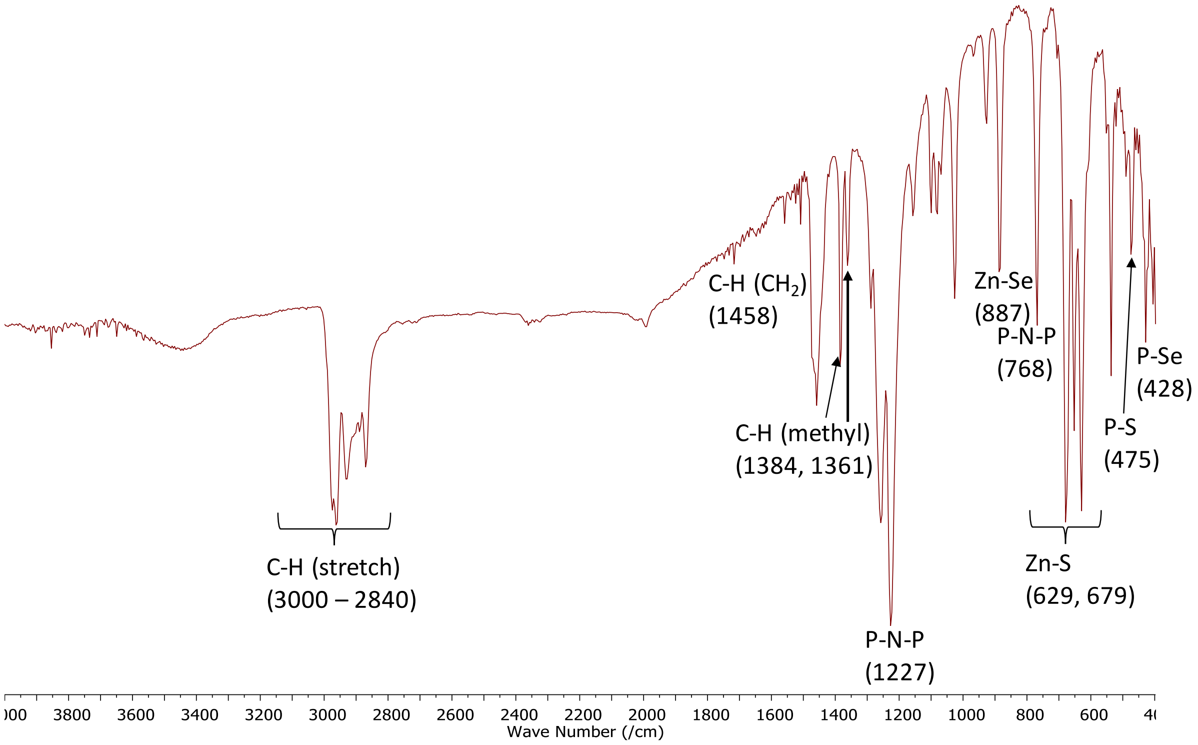

Figure S1: IR spectrum for [Zn{^{i}Pr$_2$P(S)NP(Se)^{i}Pr$_2$}$_2$] **(1).**

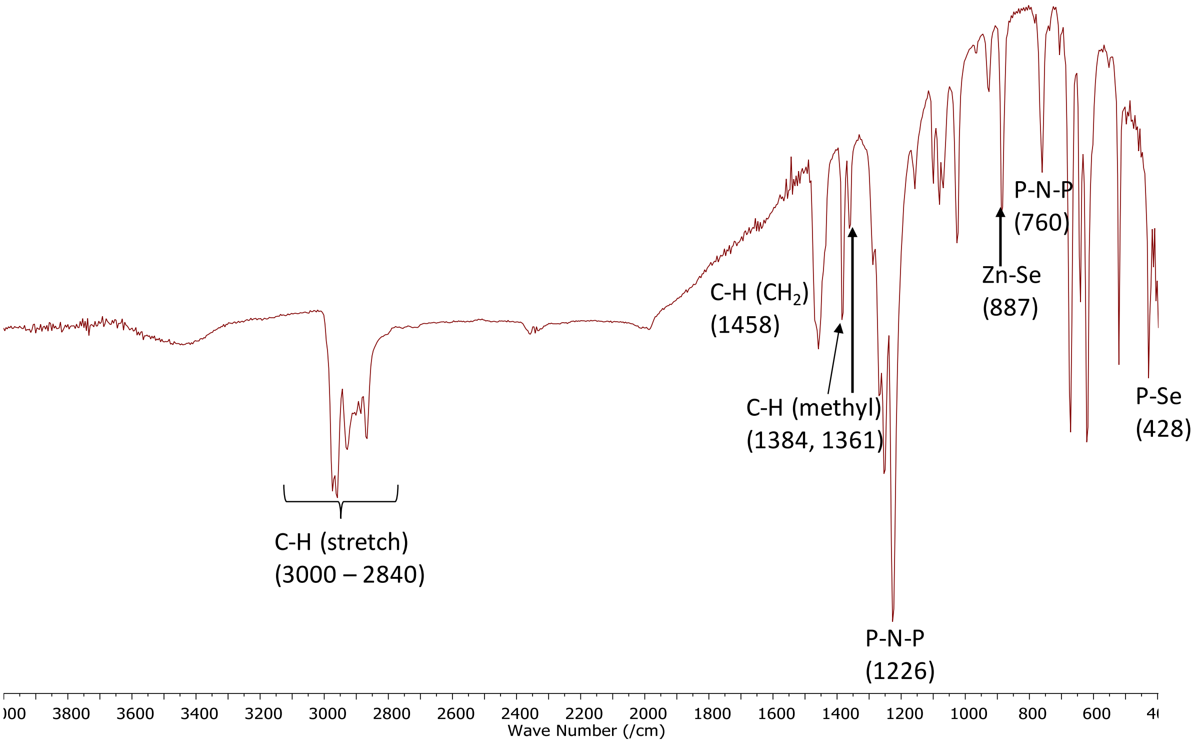

Figure S2: IR spectrum for [Zn{(SeP^{i}Pr$_2$)$_2$N}$_2$] **(2)**.

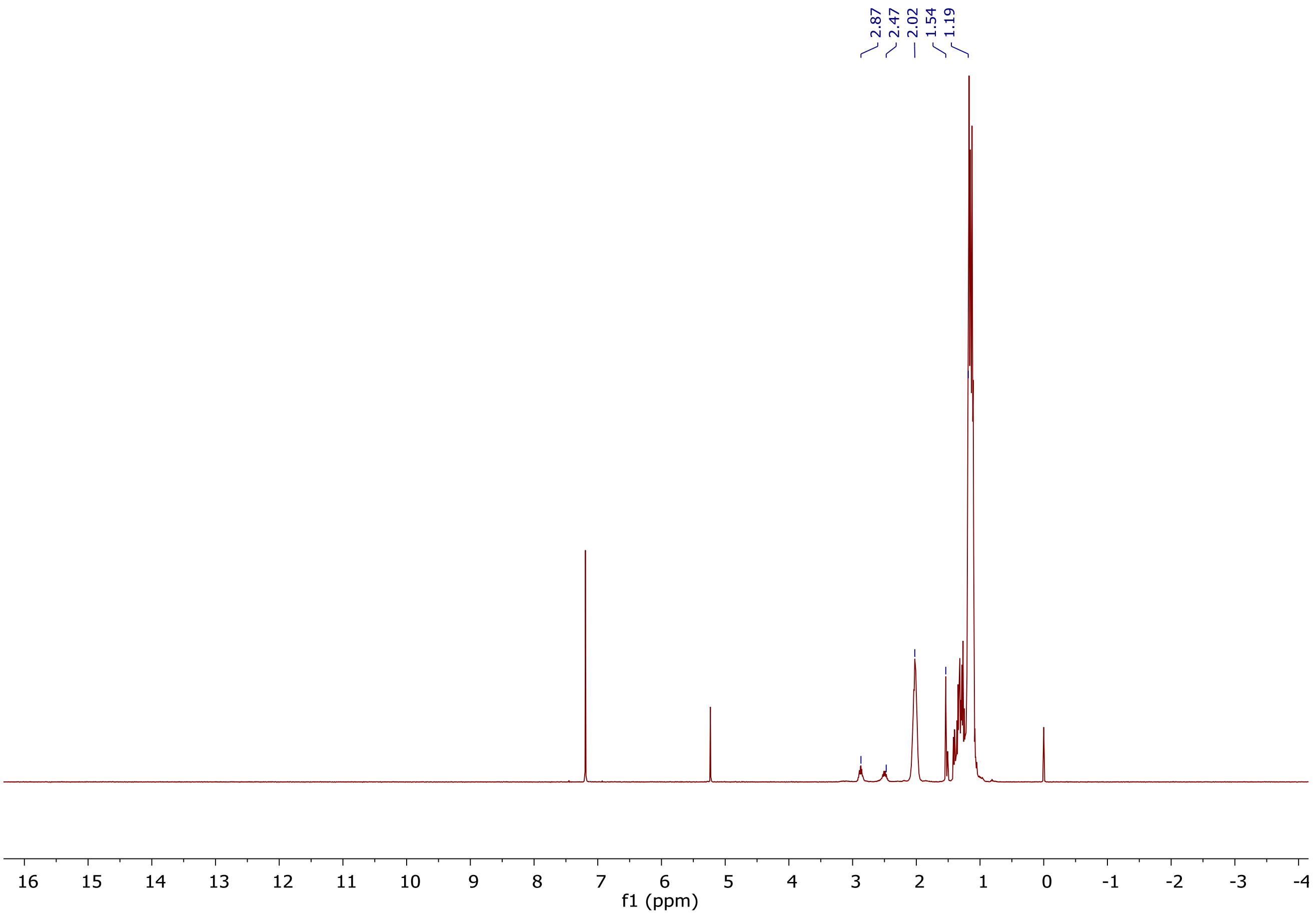

Figure S3: ^{1}H NMR spectrum for [Zn{^{i}Pr$_2$P(S)NP(Se)^{i}Pr$_2$}$_2$] **(1).**

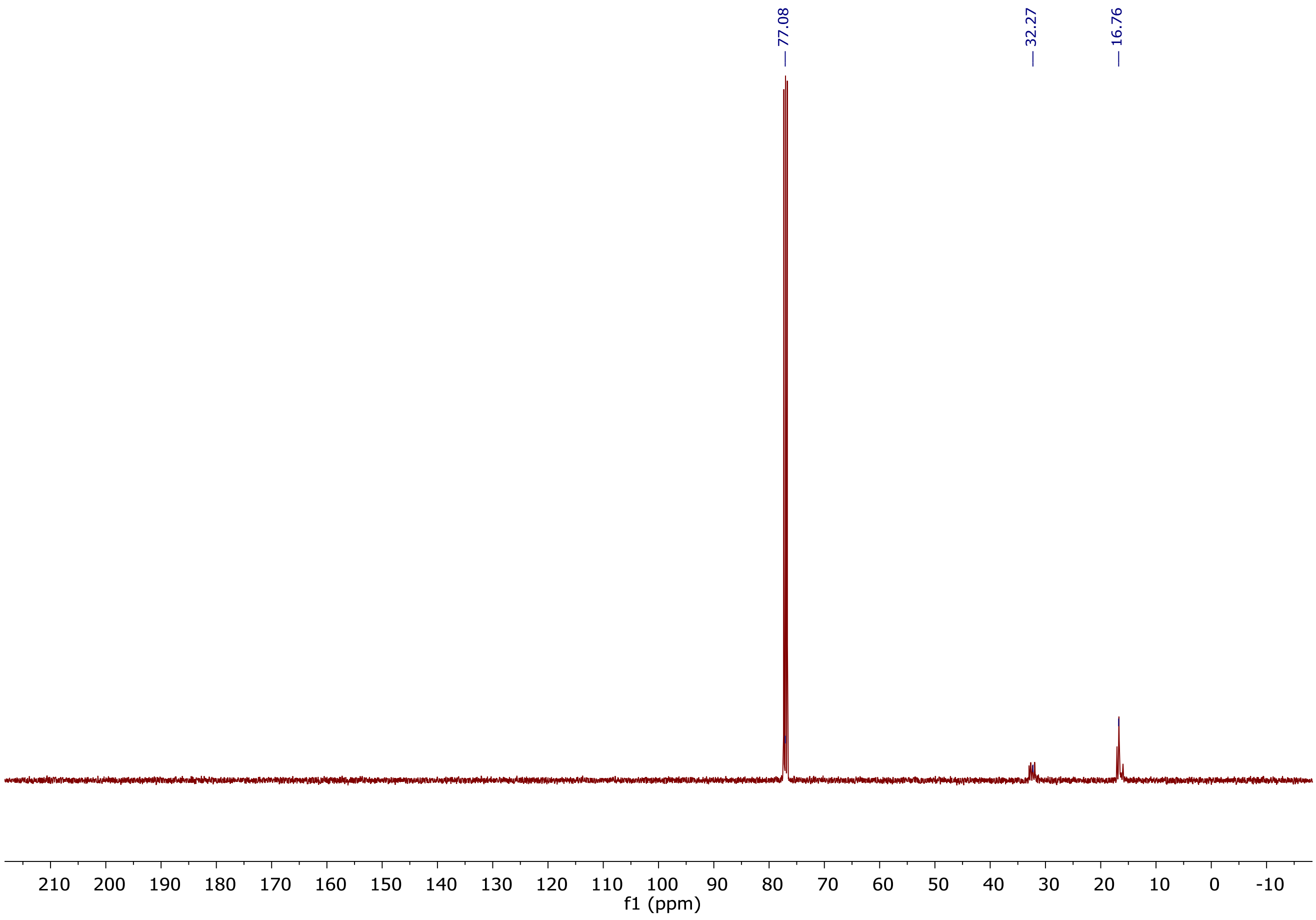

Figure S4: ^{13}C NMR spectrum for [Zn{^{i}Pr$_2$P(S)NP(Se)^{i}Pr$_2$}$_2$] **(1).**

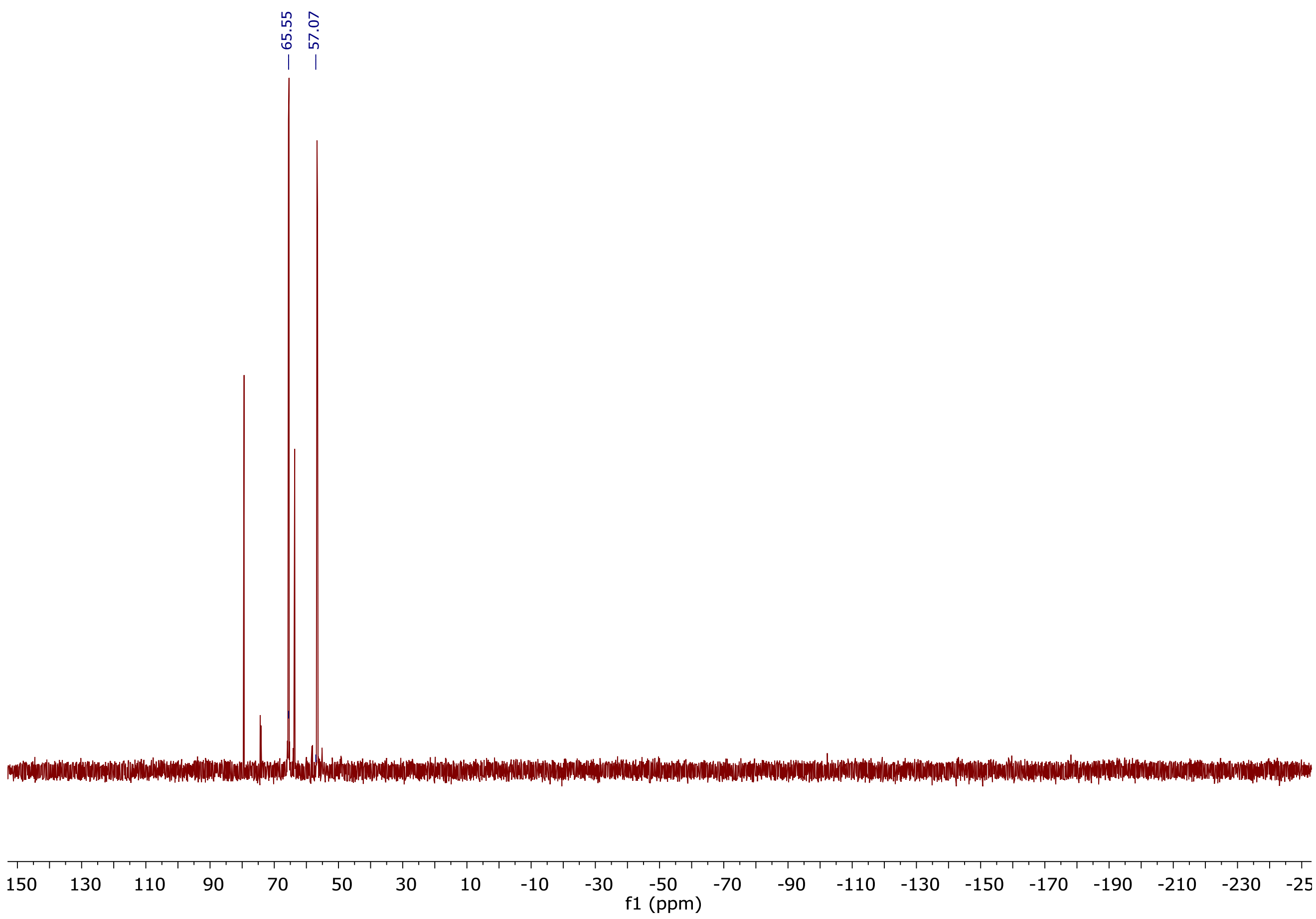

Figure S5: ^{31}P {^{1}H} NMR spectrum for [Zn{^{i}Pr$_2$P(S)NP(Se)^{i}Pr$_2$}$_2$] **(1).**

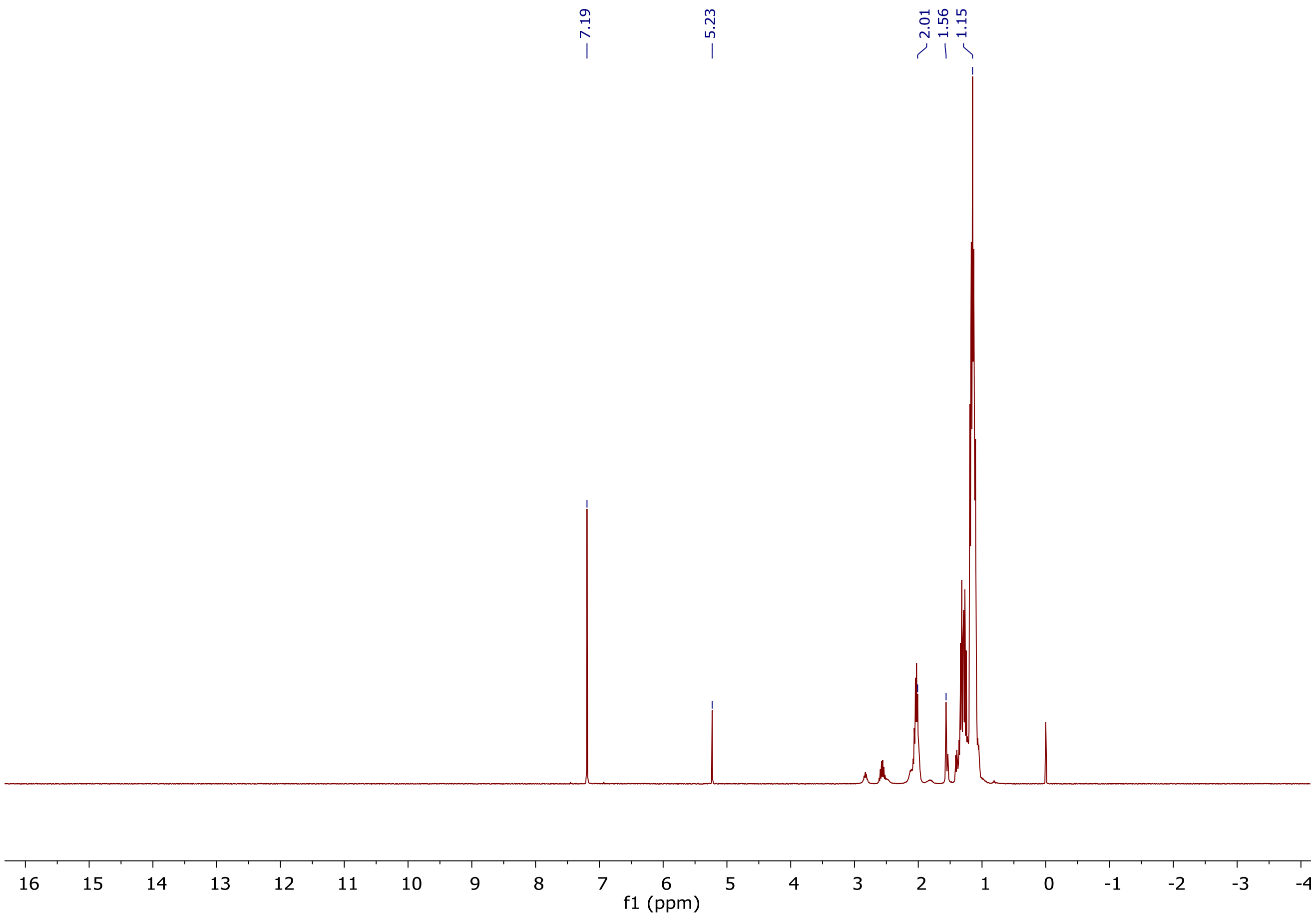

Figure S6: ^{1}H NMR spectrum for $[Zn\{(SeP^{i}Pr_2)_2N\}_2]$ **(2).**

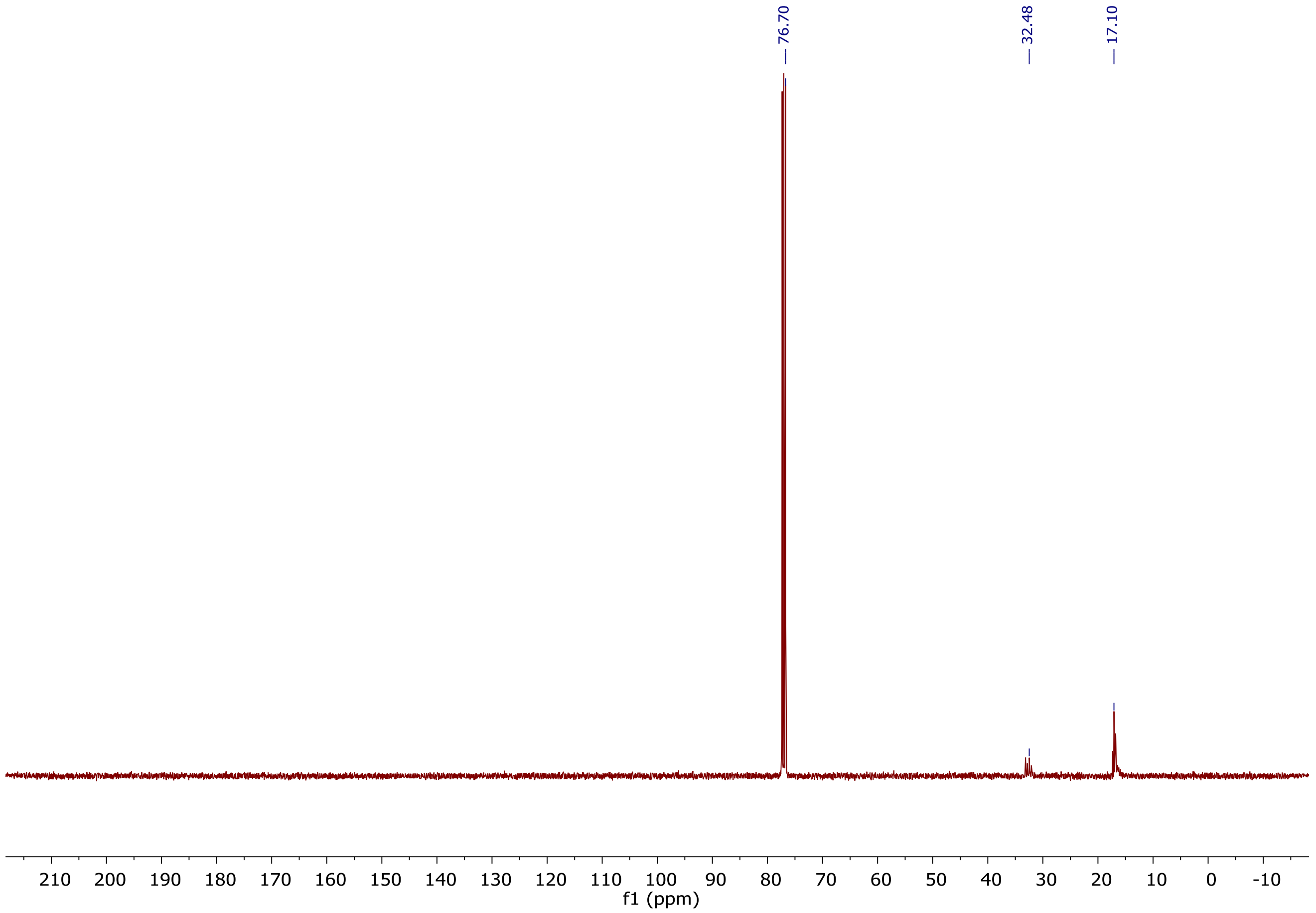

Figure S7: ^{13}C NMR spectrum for $[Zn\{(SeP^{i}Pr_2)_2N\}_2]$ **(2).**

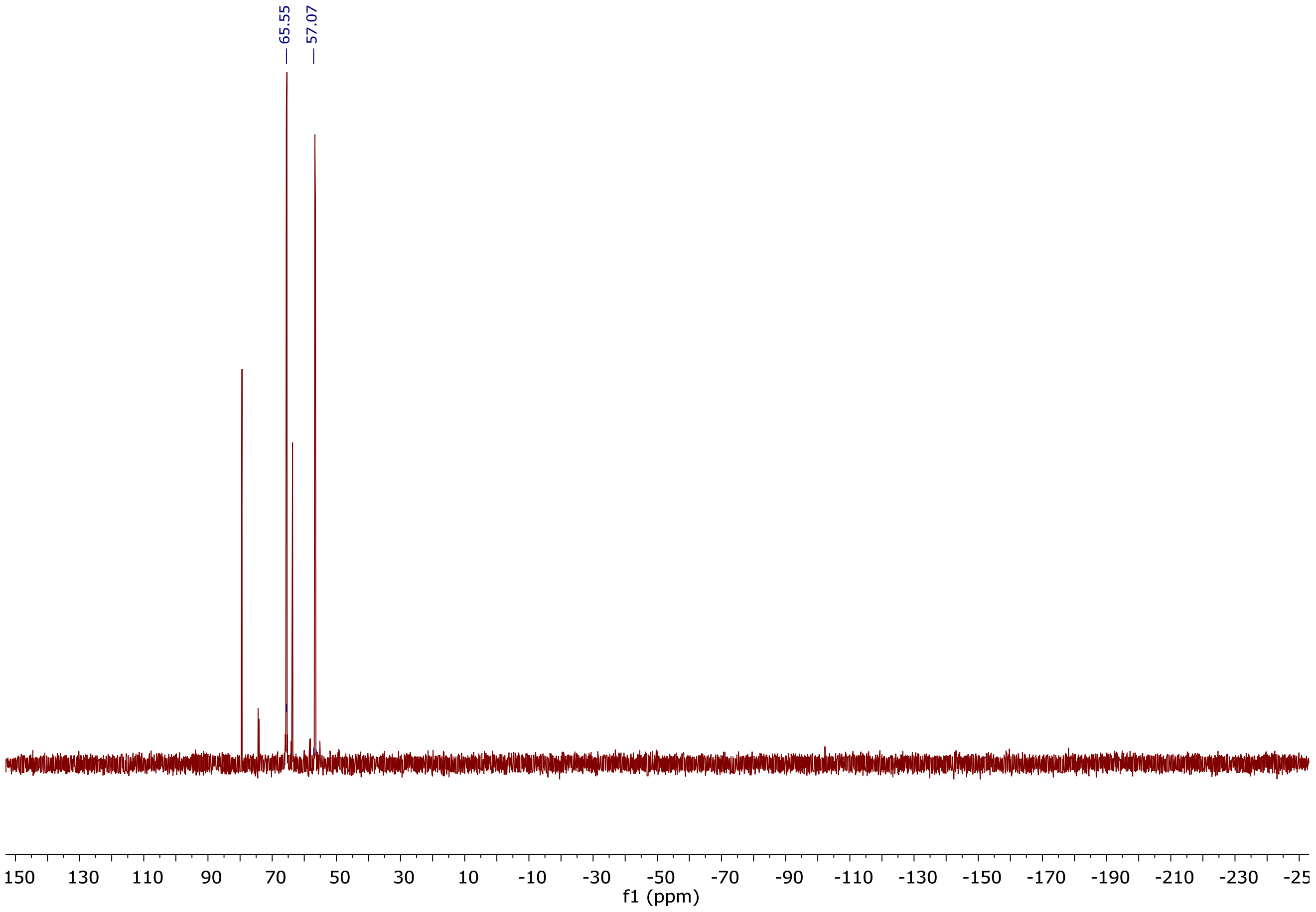

Figure S8: ^{31}P {^{1}H} NMR spectrum for [Zn{(SeP^{i}Pr$_2$)$_2$N}$_2$] **(2).**

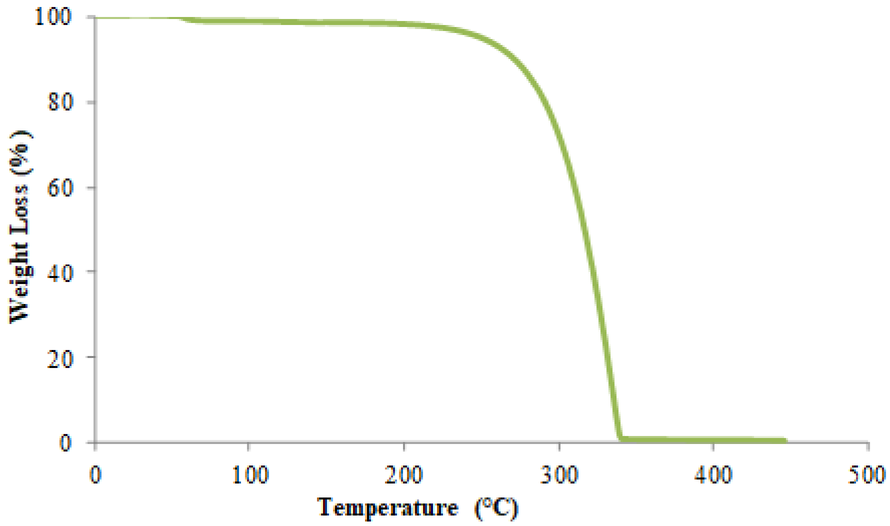

Figure S9: Thermogravimetric analysis (TGA) of [Zn{^{i}Pr$_2$P(S)NP(Se)^{i}Pr$_2$}$_2$], **(1)**.

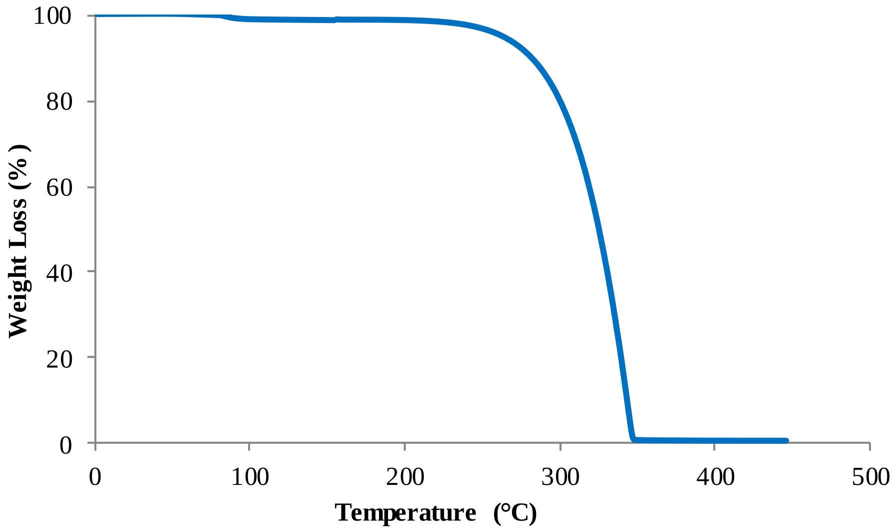

Figure S10: Thermogravimetric analysis (TGA) of [Zn{(SeP^{i}Pr$_2$)$_2$N}$_2$], **(2)**.
